# Intracerebral Human Regulatory T Cells: Analysis of CD4^+^CD25^+^FOXP3^+^ T Cells in Brain Lesions and Cerebrospinal Fluid of Multiple Sclerosis Patients

**DOI:** 10.1371/journal.pone.0017988

**Published:** 2011-03-18

**Authors:** Benedikt Fritzsching, Jürgen Haas, Fatima König, Pierre Kunz, Eva Fritzsching, Johannes Pöschl, Peter H. Krammer, Wolfgang Brück, Elisabeth Suri-Payer, Brigitte Wildemann

**Affiliations:** 1 Department of Neonatology, Center for Child and Adolescent Medicine, University Hospital of Heidelberg, Heidelberg, Germany; 2 Division of Molecular Neuroimmunology, Department of Neurology, University Hospital of Heidelberg, Heidelberg, Germany; 3 Department of Neuropathology, University Medical Center, Göttingen, Germany; 4 Department of Orthopedic Surgery, University Hospital of Heidelberg, Heidelberg, Germany; 5 Divison of Immunogenetics, German Cancer Research Center (DKFZ), Heidelberg, Germany; 6 Department of Neuropathology, University Hospital, Giessen, Germany; University of Palermo, Italy

## Abstract

Impaired suppressive capacity of CD4^+^CD25^+^FOXP3^+^ regulatory T cells (Treg) from peripheral blood of patients with multiple sclerosis (MS) has been reported by multiple laboratories. It is, however, currently unresolved whether Treg dysfunction in MS patients is limited to reduced control of peripheral T cell activation since most studies analyzed peripheral blood samples only. Here, we assessed early active MS lesions in brain biopsies obtained from 16 patients with MS by FOXP3 immunohistochemistry. In addition, we used six-color flow cytometry to determine numbers of Treg by analysis of FOXP3/CD127 expression in peripheral blood and cerebrospinal fluid (CSF) of 17 treatment-naïve MS patients as well as quantities of apoptosis sensitive CD45RO^hi^CD95^hi^ cells in circulating and CSF Treg subsets. Absolute numbers of FOXP3^+^ and CD4^+^ cells were rather low in MS brain lesions and Treg were not detectable in 30% of MS biopsies despite the presence of CD4^+^ cell infiltrates. In contrast, Treg were detectable in all CSF samples and Treg with a CD45RO^hi^CD95^hi^ phenotype previously shown to be highly apoptosis sensitive were found to be enriched in the CSF compared to peripheral blood of MS patients. We suggest a hypothetical model of intracerebral elimination of Treg by CD95L-mediated apoptosis within the MS lesion.

## Introduction

Multiple Sclerosis (MS) is considered a prototype autoimmune disease. Analysis of the autoimmune response is predominantly studied in peripheral blood of MS patients or carried out in animal models which share some similarities with MS such as EAE. Since potentially pathogenic autoreactive T cells are present in the periphery of healthy individuals [1], [2] loss of peripheral tolerance mechanisms has been suggested as a prerequisite to allow activation and migration of self-destructive inflammatory cells to the target organ. Active suppression by natural FOXP3^+^ regulatory T cells (Treg) maintains peripheral tolerance and controls autoreactive T cells [3], [4]. We and others have reported that Treg derived from peripheral blood of MS patients are functionally impaired [5]–[7] and that this Treg defect might - at least in part - reflect the reduced presence of naive Treg in MS compared to healthy individuals [8], [9]. Whereas putative autoreactive T cells are thought to exhibit resistance towards apoptosis [10]–[13] Treg from MS patients are highly sensitive to induction of CD95L-mediated apoptotic cell death as we have shown previously [14], [15]. This phenotype is not MS-specific since Treg from healthy donors exhibit similar apoptosis sensitivity and Treg turned out to be the most short-lived cells among T cells populations [15], [16]. By six-color FACS analysis we have previously identified apoptosis-prone Treg [14], [17] allowing to quantify this Treg subpopulation without further functional testing. Moreover, we have validated these assays for MS patients [6], [15]. It is a drawback, however, that most studies of Treg in MS patients are limited to the analysis of peripheral blood and do not include material derived from cerebrospinal fluid or inflamed brain tissue. Evidence from other diseases has revealed that Treg do not only inhibit activation of autoreactive T cells in the periphery but also actively suppress inflammation at the site of organ destruction [18]. Our current knowledge on Treg in the human CNS is sparse and nearly no data is available on natural FOXP3^+^ Treg distribution in the MS lesion [19].

Here, we studied the frequency of natural FOXP3^+^ cells in brain tissue derived from MS patients who underwent biopsy. We detected FOXP3^+^ cells in active lesions as well as in the cerebrospinal fluid (CSF) of treatment-naive MS patients. Whereas Treg frequencies were not decreased in the CSF compared to Treg in peripheral blood, presence of FOXP3^+^ cells in MS brain lesions was less frequent. Our finding that Treg with an apoptosis-prone phenotype are markedly enriched in the CSF from MS patients might explain this difference.

## Results

### Immunohistochemical analysis of FOXP3^+^ cells in brain lesions

**Figure 1:**
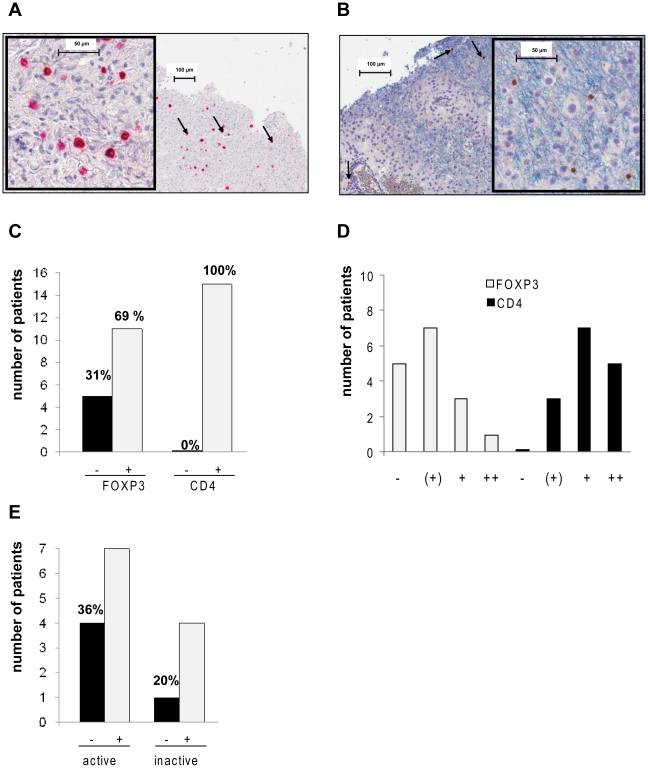
Prevalence of CD4^+^ and FOXP3^+^ cells in MS lesions. Brain biopsies of 16 MS patients were analyzed by immunohistochemistry for the presence of CD4^+^ and FOXP3^+^ cells. Sections of each biopsy were stained with CD4 mAb, FOXP3 mAb or isotype control mAbs. Whole-slide analysis by complete scanning of the section and computer-aided analysis of positive cells was performed. (**A**) Representative CD4 immunohistochemistry of MS lesion. Positive cells show red cell surface staining. Bar indicates µm scale in two different magnifications. Tissue was hematoxylin counterstained prior to analysis. (**B**) Representative FOXP3 immunohistochemistry of MS lesion. Positive cells show brown nuclear staining. Bar indicates µm scale in two different magnifications. Tissue was hematoxylin counterstained prior to analysis. (**C**) Number of patients with CD4^+^ T cells in the section (0% negative, 100% positive) and number of patients with FOXP3^+^ cells in the analyzed section (31% negative with 69% positive for FOXP3). (**D**) Number of patients grouped by their content of FOXP3^+^ cells and CD4^+^ cells respectively in the analyzed section: “-“ indicates 0 cells/mm^2^, “(+)” 0-3 cells/mm^2^, “+” >4-15 cells/mm^2^, “++” >15 cells/mm^2^. (**E**) Number of patients with (+) or without detection of FOXP3^+^ cells (-) in active or inactive MS lesions.

Inflammatory demyelinating, biopsy-derived brain lesions from 16 MS patients were analyzed by immunohistochemistry for the presence of Treg in MS lesions. We performed immunohistochemistry for FOXP3^+^ cells and CD4^+^ cells of paraffin-embedded tissue as previously established by our group [20], [21]. The specifity and sensitivity of the immunohistochemical procedure has been validated by various controls before [20], [21]. Sections of each biopsy were stained with CD4 mAb (Fig. 1 A), FOXP3 mAb (Fig. 1 B) or isotype control mAbs respectively. We established whole-slide analysis by complete scanning of the section and computer-aided analysis of positive cells. All sections from MS lesions with inflammatory infiltrates studied here revealed CD4 positive cells in the lesion (Fig. 1 C). In contrast, FOXP3^+^ cells were less frequently detectable with more than 30% of patients showing no presence of FOXP3^+^ cells at all within the whole section (Fig. 1 C, D). We repeated the staining for FOXP3 in biopsies with sufficient amount of embedded material to confirm this finding. FOXP3 positive cells were located both in perivascular areas (Fig. 1 B) as well as in the brain parenchyma. No clustering of FOXP3^+^ cells was observed within the tissue. As MS lesions show a broad histological spectrum of demyelinating and inflammatory activity, we assessed whether the presence of Treg was dependent on the underlying pathology. Our study included lesions with early active demyelination allowing the definition of the immunopathological subtype [22] as well as lesions with inflammatory activity but lack of ongoing demyelination (inactive lesions). However, paucity of FOXP3^+^ cells did not depend on the activity of the analyzed subtype (Fig. 1 E).

### Six-color FACS analysis of CSF-derived lymphocytes from treatment-naïve MS patients

We and others have previously described reduction of Treg inhibitory capacities *ex vivo* in MS [5]–[7]. We extended this study and found that (i) frequencies of naïve Treg are critical for suppressive activities measured *in vitro* and (ii) naive Treg were reduced in numbers in the peripheral circulation of the MS patients [8]. As we could not observe accumulation of FOXP3^+^ T cells in MS lesions we hypothesized that putative Treg might exhibit an apoptosis-prone phenotype once they have entered the CNS space and that rapid elimination of highly apoptosis-sensitive Treg within the brain could explain the low numbers of Treg in the MS lesion. We have recently described that MS-derived Treg exhibit similarly high apoptosis sensitivity as Treg from healthy controls [15]. Only Treg with high expression of CD45RO and CD95 commit apoptosis when they bind cell surface CD95Ligand [17].

**Figure 2:**
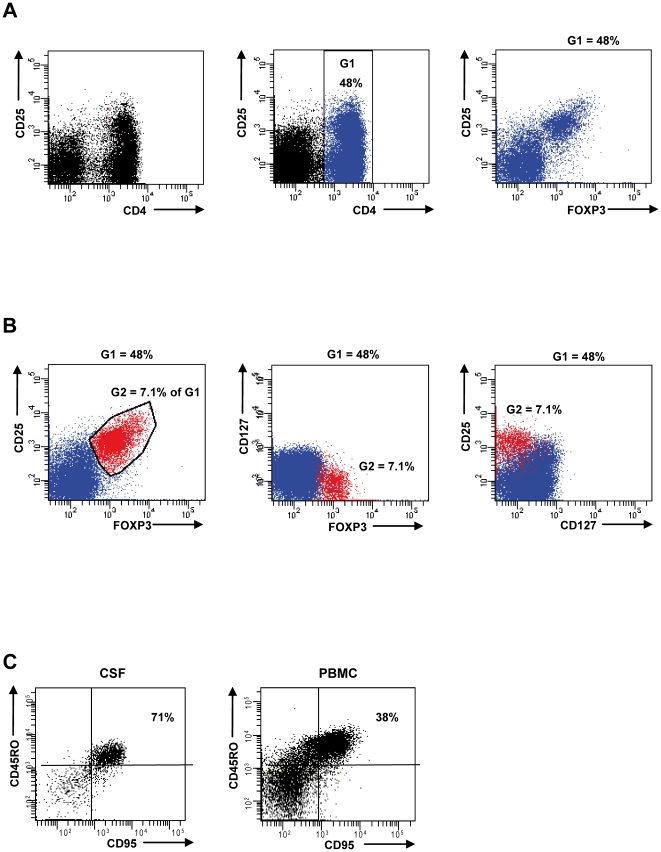
Representative six-color FACS analysis of lymphocytes derived from CSF and peripheral blood. Isolated lymphocytes were stained with monoclonal antibodies against CD4, CD25, CD127, FOXP3, CD45RO and CD95. CD4^+^ cells were gated (G1) and analyzed for FOXP3, CD127 and CD25 **(A)**. CD4^+^ cells are shown in all other dot plots of Fig. 2A, B in blue and represent 48% of peripheral lymphocytes. **(B)** CD4 positive cells with significant FOXP3 expression and low to high CD25 expression were included in gate (G2). These cells are shown in all other dot plots of Fig. 2A, B in red and represent 7.1% of CD4 positive cells. Multicolor analysis confirms that the majority of FOXP3 positive cells co-segregate with CD127^lo^CD25^lo-hi^ expression. Note: CD127 negative cells accumulate in the first few channels by conventional dot plotting. **(C)** Pairwise analysis from CSF and PBMC of a treatment-naïve MS patient. Isolated lymphocytes were stained with monoclonal antibodies against CD4, CD25, CD127, FOXP3, CD45RO and CD95. Lymphocytes were gated for CD4^+^CD25^+^CD127^lo^FOXP3^+^ cells and the percentage of CD95^hi^CD45RO^hi^ was determined.

**Figure 3:**
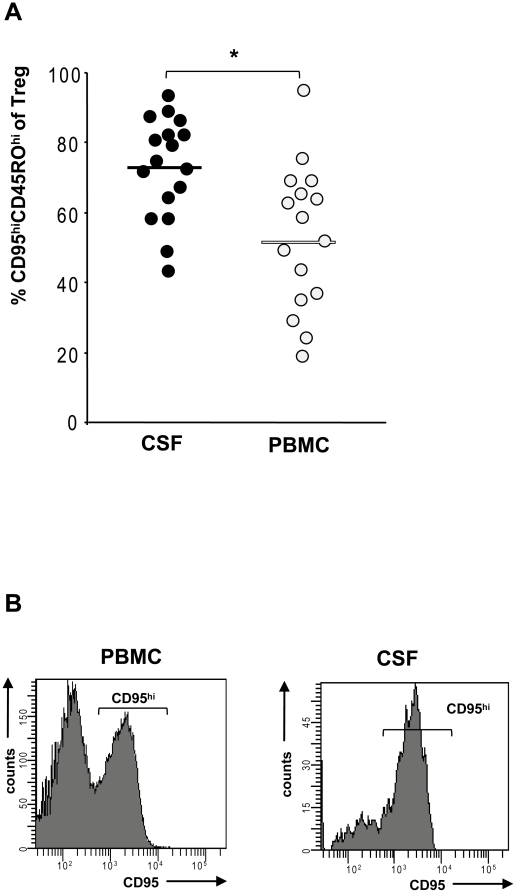
Six-color FACS analysis of lymphocytes derived from CSF and peripheral blood. Analysis of CSF and PBMC from 17 treatment-naïve MS patients. Isolated lymphocytes were stained with monoclonal antibodies against CD4, CD25, CD127, FOXP3, CD45RO and CD95. **(A)** Lymphocytes were gated for Treg and the percentage of CD95^hi^CD45RO^hi^ was determined. Treg were increased in CSF when compared to peripheral blood, * indicates p<0.05. **(B)** Expression of CD95 on Treg from CSF and PBMC derived from the same MS patient on the same day, representative figure.

Therefore, multicolor FACS analysis to determine CD4^+^CD25^+^FOXP^+^CD127^lo^CD45RO^hi^CD95^hi^ cells allows us to discriminate apoptosis-prone Treg from rather apoptosis-resistant Treg with a more naïve phenotype [17]. To this end, we pairwise isolated lymphocytes from CSF and PBMC and stained isolated cells with monoclonal antibodies against CD4, CD25, CD127, FOXP3, CD45RO and CD95 (Fig. 2). Multicolor gating revealed that CD4^+^CD25^+^FOXP3^+^ cells almost completely overlap with CD4^+^CD25^+^CD127^lo^ cells as recently described [23], [24]. As shown in Fig. 2B CD4^+^CD25^+^CD127^lo^ cells constitute a separate population. Backgating confirmed that nearly all cells correspond to FOXP3^+^ cells. Therefore, we included in some cases of CSF samples with rather low cell counts the analysis of CD4^+^CD25^hi^CD127^lo^CD45RO^hi^CD95^hi^ cells in our study. When we analyzed the percentage of CD95^hi^CD45RO^hi^ Treg in a cohort of 17 MS patients we found a significantly (p = 0.019) higher percentage of such apoptosis-prone Treg in CSF compared to peripheral blood (Fig. 2C). Likewise, CD95 expression on CSF derived Treg was higher than on Treg derived from peripheral blood resulting into a higher percentage of CD95^hi^ Treg in CSF (Fig. 3B). Functional analysis confirmed a high apoptosis sensitivity of CSF-derived Treg towards CD95L-mediated apoptosis (data not shown). As immunohistochemistry of brain biopsies revealed reduced numbers of intralesional Treg (Fig. 1) we also compared total Treg frequencies in CSF with Treg frequencies in peripheral blood. Treg were present in all CSF samples analyzed. Moreover, there was a clear tendency for higher Treg rates in CSF than in peripheral blood (mean CSF 6.8% of CD4^+^ cells vs. PBMC mean 4.8% of CD4^+^ cells).

### Migration of CD95^hi^45RO^hi^ Treg towards CSF from treatment-naïve MS patients

**Figure 4:**
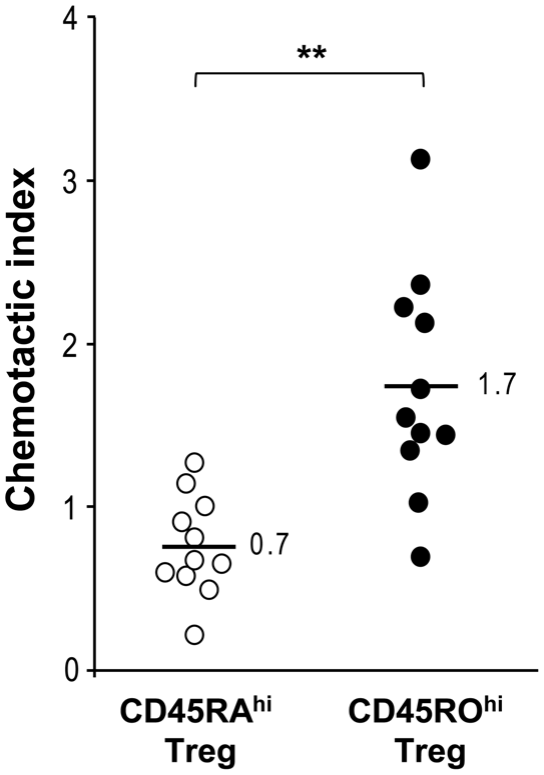
Chemotactic activities for CD45RA^hi^ Treg and CD45RO^hi^ Treg subsets. Chemotactic indices (CI) of Treg from a healthy donor towards CSF supernatants of 11 MS patients as determined by transwell migration assays. Original input PBMC and migrated cells were stained for CD4, CD25, FOXP3, CD95, CD45RO, and CD45RA, and CIs were calculated by dividing percentages of Treg subsets in CD4^+^ T-cells of migrated cells by percentages of Treg in CD4^+^ T-cells in original input PBMCs. Symbols indicate CIs of individual patients. Means are indicated, ** indicates p<0.001.

Alterations in migration properties of Treg could explain low numbers of Treg in the active MS lesion. To address this question we performed transwell migration experiments to analyze chemotaxis of Treg towards CSF from MS patients. We specifically determined the migration capacity of CD95^hi^CD45RO^hi^CD45RO^hi^ Treg versus its CD45RA^hi^ naïve counterpart. In accordance with the accumulation of CD95^hi^CD45RO^hi^CD45RO^hi^ Treg in patient derived CSF, this subset exhibited a significantly higher chemotactic activity (CI-CD45RA^lo^CD45RO^hi^: 1.71±0.53, CI-CD45RA^hi^CD45RO^lo^: 0.74±0.24, p<0.001; Fig. 4).

## Discussion

Multiple sclerosis is associated with a functional defect of natural Treg as we and others have reported [5]–[7]. Most studies are limited to the analysis of peripheral blood and show no numeric Treg abnormality but reduced *in vitro* suppressive capacity of CD4^+^CD25^hi^ Treg. However, Treg perform their inhibitory function in the lymphoid and other target organs *in vivo*. We and others could detect Treg accumulation at sites of inflammation in several human inflammatory diseases [20], [21], a process potentially also initiated by cancer cells to attract Treg into human tumor tissue [25]. However, no studies of Treg in early MS lesions from brain biopsies of treatment-naïve patients with active MS are currently available. Knowledge regarding quantities and phenotype of Treg in the CSF is also scarce. Previous analysis of Treg in CSF was performed by detecting CD27^+^ or CD25^hi^ cells [6], [7], [23] but no data is available on the complete Treg population defined by FOXP3 and CD127 expression levels and their detailed phenotypic characterization is missing. Nevertheless, previous studies of such ill-defined subpopulations show accumulation of Treg in the CSF when compared to peripheral blood [7], [23].

Here, we studied the frequencies of natural FOXP3^+^ T cells in brain lesions derived from 16 RR-MS patients who underwent brain biopsy. We detected FOXP3^+^ T cells mainly in active lesions. Further, Treg were abundant in the CSF of treatment-naïve MS patients and our findings confirm the trend for enhanced frequencies compared to peripheral blood within the same patient (pairs from CSF and peripheral blood samples from 13 MS patients were analyzed, CSF samples were derived from 17 MS patients in total). Treg migrate easily into the CNS compartment as we have previously published although extensive proliferation of Treg in the CNS cannot be ruled out [26]. Similarly, other immunomodulatory T cells with expression of HLA-G were reported to accumulate in the CSF [27]. These observations are important, since novel drugs such as natalizumab aim to inhibit the transfer of deleterious T cells into the CNS. However, no data is available to clarify if application of such agents might not only inhibit migration of autoreactive effector T cells, but might also interfere with Treg migration into MS brain lesions [28].

Whereas Treg rates showed a tendency for higher frequencies in CSF when compared to Treg frequencies in peripheral blood, FOXP3^+^ T cells were hardly present in the MS brain lesion. 70% percent of early MS lesions showed low FOXP3^+^ T cell infiltration in both perivascular areas and regions without blood vessels. Clusters between FOXP3^+^ and FOXP3^−^ T cells were not observed. FOXP3^+^ cells were both present in regions with high CD4 numbers as well as in regions with low CD4 numbers within the MS lesion. In the remaining 30% of patients we were unable to detect any FOXP3 positive cells, even in the presence of CD4^+^ cells in the brain biopsies. A low number of FOXP3^+^ T cells in the MS lesion is in line with available data of post-mortem brain material from 14 MS patients [19]. Although several groups show CD4^+^ infiltration as a typical feature of MS lesions [19], [29]–[32] FOXP3^+^ cells were not detectable in any of the post mortem specimens analyzed [19]. However, it cannot be ruled out that post mortem brain material from MS patients with 10 or many more years disease duration and/or immunomodulating therapy might differ significantly from biopsies obtained from early MS lesions from newly diagnosed treatment-naïve MS patients.

Determination of precise Treg frequencies within the MS lesion appears rather problematic. Transient upregulation of FOXP3 in non-regulatory T cells has been shown by several laboratories. Moreover, multimarker analysis as used by flow cytometry cannot be applied (i.e. CD127, CD62L, FOXP3, CD25, CD4) to detect Treg by immunohistochemistry. FOXP3^+^ Tcon are currently thought to have a lower expression level of FOXP3 than Treg [45]. Of note, immunohistochemical analysis of cell sorted CD4^+^ subpopulations revealed that anti-FOXP3 immunohistochemistry as used here did only detect CD4^+^CD25^+^ cells with high FOXP3 expression (data not shown). Therefore, when taking into account the possibility of FOXP3^+^ Tcon being present within lesions, Treg frequencies might be even lower. This would strengthen our hypothesis of Treg paucity in the MS lesion.

A low number of T cells with immunosuppressive function is not a general feature of the human brain. Similar to EAE in mice Treg accumulate in brain parenchyma surrounding human brain tumors [33] and HLG^+^ regulatory T cells detectable in MS plaques [27]. We hypothesize that a dysregulated Treg homeostasis might account for low intralesional Treg numbers in MS. CD95L mediated apoptosis is a major mechanism to control Treg numbers as we could show in a previous study [14], [17]. Thymus-derived naïve Treg are resistant towards CD95L mediated apoptosis. Following Treg activation, cell surface expression of CD95 is rapidly upregulated concomitantly with an increased sensitivity towards CD95L mediated cell death. Such preactivated Treg are apoptosis-prone and constitute the majority of CD25^hi^FOXP3^+^ Treg in the peripheral circulation of healthy adults. In contrast to conventional T cells, Treg do not persist as apoptosis-resistant memory cells. High apoptosis sensitivity renders Treg an extremely short-lived population among T cells [16]. *In vivo*, Treg seem even more sensitive than activated conventional T cells towards CD95L [34]–[36]. Our previous studies of apoptosis sensitivity from various Treg subpopulations revealed that Treg with high expression of CD95 and CD45RO consistently die when challenged with CD95L *in vitro* [14], [15], [17]. By analyzing the percentage of these apoptosis-prone CD95^hi^CD45RO^hi^ Treg in MS patients we found a significant enrichment of these cells in CSF (Fig. 3). Since we have previously shown that peripheral blood Treg from treatment-naïve MS patients and healthy individuals have a similar susceptibility to undergo apoptosis [6], we exclude the possibility that genetic alterations might lead to an altered apoptosis sensitivity of Treg in MS. Thus, we consider it very likely that CD95^hi^CD45RO^hi^ Treg of the CSF from MS patients are highly apoptosis-prone. Functional analysis of the whole CSF lymphocyte fraction confirmed a high apoptosis sensitivity of CSF-derived Tregs towards CD95L-mediated apoptosis (data not shown). In contrast, percentages of total apoptotic cells in CSF were rather low. This coincided with low numbers of recently activated Tcon expressing CD69 and CD38 (data not shown). In the MS lesion, expression levels of CD95L and sensitivity towards CD95L mediated apoptosis might increase after TCR restimulation. Interaction with non-immune cells might further modify intralesional cell death rates. Elimination of Treg by CD95L could occur not only by cell contact with CD95L positive Tcon but also by contact with other CD95L expressing CNS cells (i.e. endothelium) within the MS lesion. However, due to very low numbers of FOXP3^+^ T cells, we could not determine cell death rates by immunohistochemistry.

Alterations in migration properties of Treg could also explain low numbers of Treg in the active MS lesion. In vitro, human Treg seem to migrate even faster than Tcon. Moreover, under inflammatory conditions Treg from MS patients migrate to a similar extent as Treg from healthy individuals [44]. Here, we analyzed Treg migration towards CSF from MS patients with active MS and did not observe a failure of Treg migration. Of note, migration assays revealed, that CD95^hi^CD45RO^hi^ Treg are more strongly attracted by patient derived CSF compared to CD45RA^hi^ Treg consistent with their accumulation in CSF during disease activity.

In summary, we observe active migration of CD95^hi^CD45RO^hi^ Treg towards CSF of MS patients, accumulation of CD95^hi^CD45RO^hi^ Treg in CSF and high apoptosis-sensitivity of CSF-derived Treg from MS patients. We detect low numbers of FOXP3 cells in MS lesions, but cannot proof enhanced intralesional Treg elimination.

In a hypothetical model we suggest (i) peripheral Treg activation followed by (ii) migration into the cerebrovascular circulation. Transmigration of Treg into MS lesions might be accompanied by (iii) Treg restimulation of CNS cells with antigen presenting function. Due to several rounds of TCR stimulation Treg are sensitive to undergo apoptosis. Constant elimination of Treg in the MS lesion could be the reason for the low numbers of intralesional Treg in our study.

Several other factors might also render Treg susceptible to cell death in the autoimmune lesion, e.g. IL-2 deprivation. It was recently demonstrated that Tregs in pancreatic tissue of NOD mice are prone to apoptosis because of insufficient levels of IL-2 [37]. Likewise, we have shown previously that Treg are prone to Il-2 deprivation death in MS [15]. Whether Il-2 levels are decreased in the MS lesion remains to be determined. Collectively, we favor a model of enhanced Treg turnover in the target organ during active MS, which might influence the homeostatic composition of the Treg pool.

Our findings shed a new light on the initial finding of reduced Treg inhibitory function in MS patients. Taking the available data together the following picture emerges. The suppressive capacity of the highly purified *in vivo* preactivated CD25^hi^CD45RO^hi^ Treg subpopulation is normal in RR-MS patients [8], [9], [38]. In contrast, alterations within the naïve Treg pool possibly related to reduced thymic output of naïve Treg in MS might explain the reduced suppressive function initially observed in the total Treg population in RR-MS [8], [9]. Enhanced Treg turnover in the MS lesion as reported here might further skew the balance of naïve and activated Treg so that activated CD95^hi^CD45RO^hi^ Treg become the prevalent population.

## Methods

### Patients

The study included 33 patients (16 with CNS biopsy, 17 with CSF samples) with definite relapsing remitting MS according to the Poser criteria [39]. Brain biopsies from 16 patients with histopathologically confirmed MS were obtained from the Department of Neuropathology, University Medical Center Göttingen. All patients underwent brain biopsy to rule out other CNS diseases such as lymphoma, toxoplasmosis or vasculitis.

### Patients: brain biopsies

By using conventional and immunocytochemical stainings each MS case revealed characteristics of inflammatory demyelination of the CNS consistent with Mutilpe Sclerosis [40], [41]. The lesions were then categorized according to their stage of demyelinating activity [42]. Here, lesions with early active (EA) demyelination show numerous infiltrating cells including foamy macrophages containing myelin degradation products of all major myelin proteins (MBP, PLP, MAG, MOG, CNPase). Inactive lesions (IA) demonstrate complete demyelination, and macrophages do not reveal signs of cytoplasmatic myelin protein degradation. However inactive demyelinating lesions are still inflammatory active.

According to Lucchinetti and Brück lesions with early active demyelination reveal either of four different immunopathological subtypes of multiple sclerosis, indicating pathogenetic heterogeneity [22]. Patterns (IP) I and II are characterized by a marked T cell/macrophage-associated demyelination, with (IP II) or without (IP I) additional immunoglobulin and complement depositions at sites of active myelin destruction, respectively. These two patterns belong to the so-called autoimmune-mediated subtypes. In contrast pattern III and IV reveal marked oligodendropathology. Here, pattern III lesions show signs of a distal oligodendrogliopathy with detection of apoptotic oligodendrocytes, and a distinct loss of MAG or CNPase. Pattern IV, however, is a very rare subtype which is restricted to primary progressive MS and associated with primary oligodendrocyte degeneration.

In this study, 16 MS biopsies were enrolled. Eleven samples revealed early active demyelination (four pattern I lesions, five pattern II specimens, and two pattern III samples, but no pattern IV biopsies). Five additional cases revealed inactivity with respect to demyelination.

### Patients: CSF and peripheral blood samples

CSF samples and peripheral blood specimens were derived pairwise from 17 unrelated patients at the same day. All 17 patients were newly diagnosed with MS according to the revised McDonald or Poser criteria [39], [43], had clinically active disease and had not yet received treatment with corticosteroids or immunomodulatory agents. Patients had mean disease duration of 14 months, a mean Expanded Disability Status Score (EDSS) of 2.4, and had previously experienced an average of two relapses. The protocol was approved by the University of Heidelberg ethics committee and all individuals gave written informed consent.

### Antibodies and reagents

The monoclonal antibody (mAb) against CD4, CD127, CD45RO, CD95 and isotype controls were obtained from BD Pharmingen (Heidelberg, Germany), αCD25 mAb from Miltenyi Biotech (Auburn, CA) and αFOXP3 mAb (clone 236A/E7) from eBioscience (San Diego, USA).

### Immunohistochemistry

Brain biopsies were fixed in 4% paraformaldehyde and embedded in paraffin. Hematoxylin and eosin (H&E), CD4, FOXP3 and mouse IgG1 isotype control stainings were performed as previously described by our group [20], [21]. Whole slide analysis was performed with an aperio scanner to allow computer-aided analysis of the sections. All specimens were blinded and analyzed by two independent investigators. Standardized analysis was performed.

### Six-color FACS analysis

In most cases (13 pairs) pairwise analysis of peripheral blood derived lymphocytes and CSF-derived lymphocytes was performed. Lymphocyte preparation, immunostaining and FACS analysis were carried out immediately after clinical sample acquisition to prevent preanalytic alterations. To this end, peripheral blood mononuclear cells (PBMCs) were obtained from 20 ml EDTA-blood by density gradient centrifugation using Ficoll/Hypaque (Biochrom, Berlin, Germany). Likewise, 0.5 to 4 ml CSF was centrifuged to isolate mononuclear cells. Cells were stained with monoclonal antibodies (mAb) against CD4, CD127, CD45RO, CD95, CD25 and FOXP3 or isotype control mAbs and FACS analysis was performed at a LSR II flow cytometer as previously described [17], [21]. Freezing and thawing was avoided since control experiments showed significant alterations in CD95^hi^CD45RO^hi^ cell numbers when cells were stored in the freezer previous to immunostaining (data not shown).

### Migration assay

Migration activities of Treg subsets were assessed by using a transwell assay as previously described [26]. In short, 10^6^ freshly isolated PBMCs obtained from one healthy standard donor were suspended in 200 µl RPMI and transferred into the upper chambers of 6.5-mm diameter, 5.0 µm pore-size polycarbonate membrane filter transwell plates (Costar Corning, Cambridge, MA). CSF supernatants were added to the lower chamber. After 4 hours at 37°C, migrated cells were collected and FACS-analyzed. Chemotactic indices (CI) were defined as the ratio of percentages of the distinct Treg subpopulation in migrated PBMCs and of original PBMCs.
